# GM-CSF receptor/SYK/JNK/FOXO1/CD11c signaling promotes atherosclerosis

**DOI:** 10.1016/j.isci.2023.107293

**Published:** 2023-07-11

**Authors:** Daisuke Tsukui, Yoshitaka Kimura, Hajime Kono

**Affiliations:** 1 Department of Internal Medicine, Teikyo University School of Medicine, Tokyo 173-8605, Japan; 2 Department of Microbiology and Immunology, Teikyo University School of Medicine, Tokyo 173-8605, Japan

**Keywords:** Molecular biology, Immunology

## Abstract

Atherosclerosis complicates chronic inflammatory diseases, such as rheumatoid arthritis and systemic lupus erythematosus, suggesting that a shared physiological pathway regulates inflammatory responses in these diseases wherein spleen tyrosine kinase (SYK) is involved. We aimed to identify a shared therapeutic target for atherosclerosis and inflammatory diseases. We used *Syk*-knockout atherosclerosis-prone mice to determine whether SYK is involved in atherosclerosis via the inflammatory response and elucidate the mechanism of SYK signaling. The *Syk*-knockout mice showed reduced atherosclerosis *in vivo*, and macrophages derived from this strain showed ameliorated cell migration *in vitro*. CD11c expression decreased on *Syk*-knockout monocytes and macrophages; it was upregulated by forkhead box protein O1 (FOXO1) after stimulation with granulocyte-macrophage colony-stimulating factor (GM-CSF), and c-Jun amino-terminal kinase (JNK) mediated SYK signaling to FOXO1. Furthermore, FOXO1 inhibitor treatment mitigated atherosclerosis in mice. Thus, GM-CSF receptor/SYK/JNK/FOXO1/CD11c signaling in monocytes and macrophages and FOXO1 could be therapeutic targets for atherosclerosis and inflammatory diseases.

## Highlights

• *Syk*^del/del^ mice exhibit reduced atherosclerosis and macrophage accumulation
• SYK promotes macrophage cell migration via upregulated CD11c expression
• GM-CSF receptor/SYK/JNK/FOXO1/CD11c signaling occurs in macrophages
• FOXO1 inhibitor decreases CD11c expression and ameliorates atherosclerosis in mice

## Graphical abstract

**Figure:**
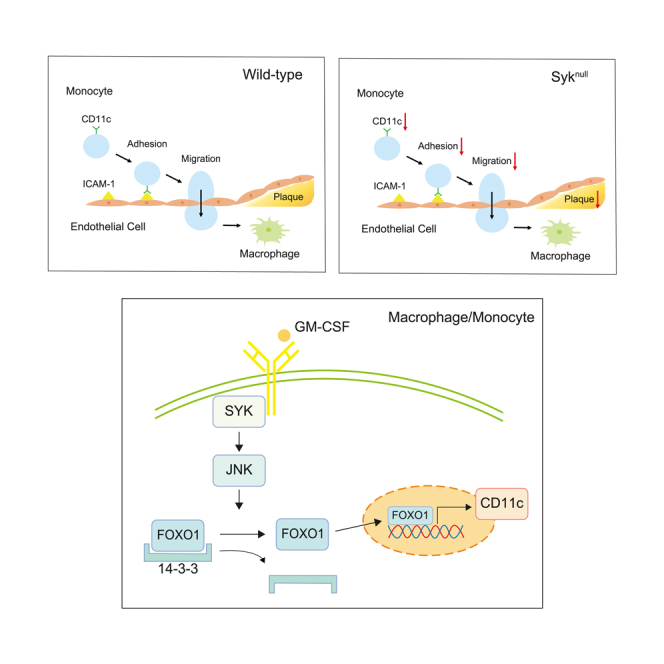

## Introduction

Chronic inflammation promotes the development of atherosclerosis and cardiovascular disease.^1^ Although hypercholesterolemia—in particular, high levels of low-density lipoprotein cholesterol (LDL-C)—is a significant risk factor for atherosclerosis,^2^ lipid-lowering therapy, such as statin administration, does not completely avert the risks of cardiovascular disease.^3^ There is a residual risk owing to factors, such as triglyceride, apolipoprotein B-100, and inflammation levels.^1^^,^^4^^,^^5^ Previous studies have reported that inflammation is also involved in the development of atherosclerosis; for instance, the level of high-sensitivity C-reactive protein (CRP), a well-known serum inflammation marker in clinical use, can predict cardiovascular events, including myocardial infarction and stroke, and the need for a coronary revascularization procedure.^6^^,^^7^

Inflammation plays a pivotal role in atherosclerotic development. LDL-C-induced hyperlipidemia results in the accumulation of LDL-C in vessel walls. Subsequently, LDL-C is oxidized and engulfed by macrophages, which secrete inflammatory cytokines, resulting in cellular infiltration from the bone marrow.^8^ The process of cellular infiltration into a lesion is common in chronic systemic inflammatory diseases, such as rheumatoid arthritis (RA) and systemic lupus erythematosus (SLE), which are considered high-risk factors for cardiovascular events.^9^^,^^10^ Furthermore, these disease activity scores correlate with an exacerbation of atherosclerotic development.^11^^,^^12^

Herein, we focused on spleen tyrosine kinase (SYK), which is a non-receptor tyrosine kinase expressed in hematopoietic cells and regulates the downstream signaling of various cell surface receptors,^13^ for the following reasons. A genome-wide association study revealed that patients with vascular dementia harbor a risk-related variant of the *Syk* gene.^14^ In addition, SYK is highly expressed in human atherosclerotic plaques,^15^ whereas an SYK inhibitor ameliorates RA and SLE in a murine model.^16^^,^^17^ Although SYK carries out several functions, such as inflammatory cytokine production, phagocytosis of oxidized LDL, and cell differentiation,^18^^,^^19^^,^^20^^,^^21^^,^^22^ its involvement in the shared pathophysiology between atherosclerosis and chronic systemic inflammatory diseases remains unclear.

Therefore, we used *Syk*-knockout mice to identify common pathological mechanisms between atherosclerosis and chronic systemic inflammatory diseases to discover new therapeutic targets to treat both conditions.

## Results

### SYK promotes atherosclerosis and macrophage invasion on atherosclerotic plaques

**Figure 1:**
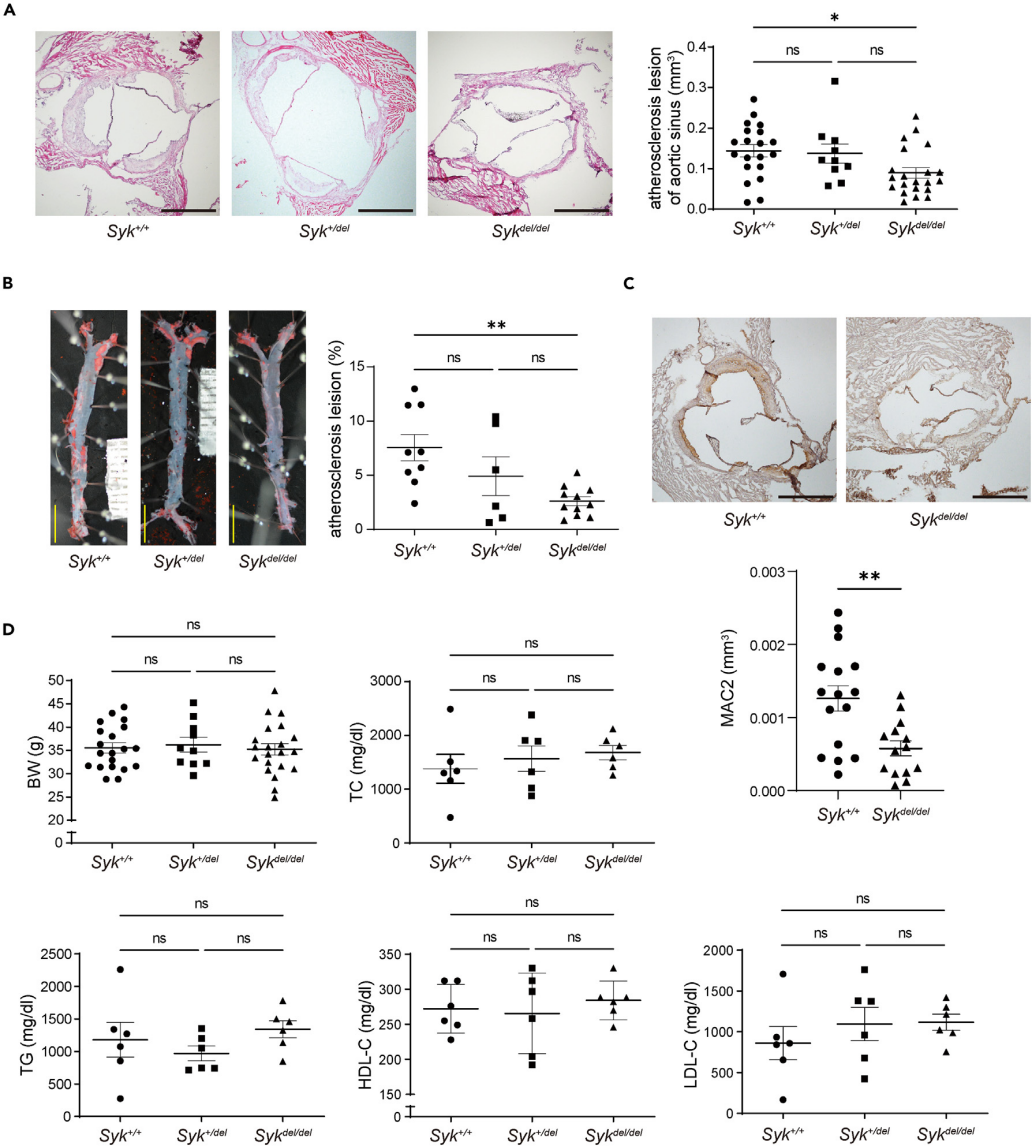
SYK exacerbates atherosclerosis and the recruitment of macrophages in atherosclerotic lesions without affecting lipid profile (A) Representative hematoxylin-eosin staining of the aortic sinus (n = 10–21; Kruskal-Wallis test; Dunn’s multiple comparison test; scale bar: 500 μm). (B) Oil Red O staining of the aorta (n = 6–11; one-way ANOVA; Dunnett’s T3 multiple comparisons test; scale bar: 5 mm) and (C) Mac-2 staining (macrophage marker) of the aortic sinus (n = 14–16; unpaired *t* test; scale bar: 500 μm). (D) Each graph shows body weight (BW), serum levels of total cholesterol (TC), triglyceride (TG), HDL-cholesterol (HDL-C), and LDL-cholesterol (LDL-C) (n = 10–21 for BW; n = 6 per group for other parameters; one-way ANOVA). Data are shown as mean ± SEM. ∗p < 0.05, ∗∗p < 0.01; Ns: not significant. *Syk* = spleen tyrosine kinase.

First, we generated *Syk*^*flox/flox*^*Rosa26CreER*^*(T2)+/+*^*Ldlr*^*−/−*^ mice. The *Syk* gene was systemically knocked out using tamoxifen. *Syk* deletion from each organ was confirmed using PCR and western blotting (Figures S1A and S1B). *Ldlr* knockout was also confirmed using PCR (Figure S1C). Mice were fed a high-fat diet for 16 weeks for atherosclerosis induction. The cumulative atherosclerotic area in the aortic sinus and the aorta in *Syk*^*del/del*^ mice was considerably smaller than that in *Syk*^*+/+*^ mice (Figures 1A and 1B). Next, we measured the macrophage area of the atherosclerotic plaque as macrophages play a central role in atherosclerosis.^8^ The macrophage area of *Syk*^*del/del*^ mice was smaller than that of *Syk*^*+/+*^ mice (Figure 1C). However, both groups showed no significant differences in body weight and serum cholesterol levels (Figure 1D). These results indicate that SYK promoted atherosclerosis regardless of hypercholesterolemia.

### SYK regulates cell migration via CD11c expression on macrophages

**Figure 2:**
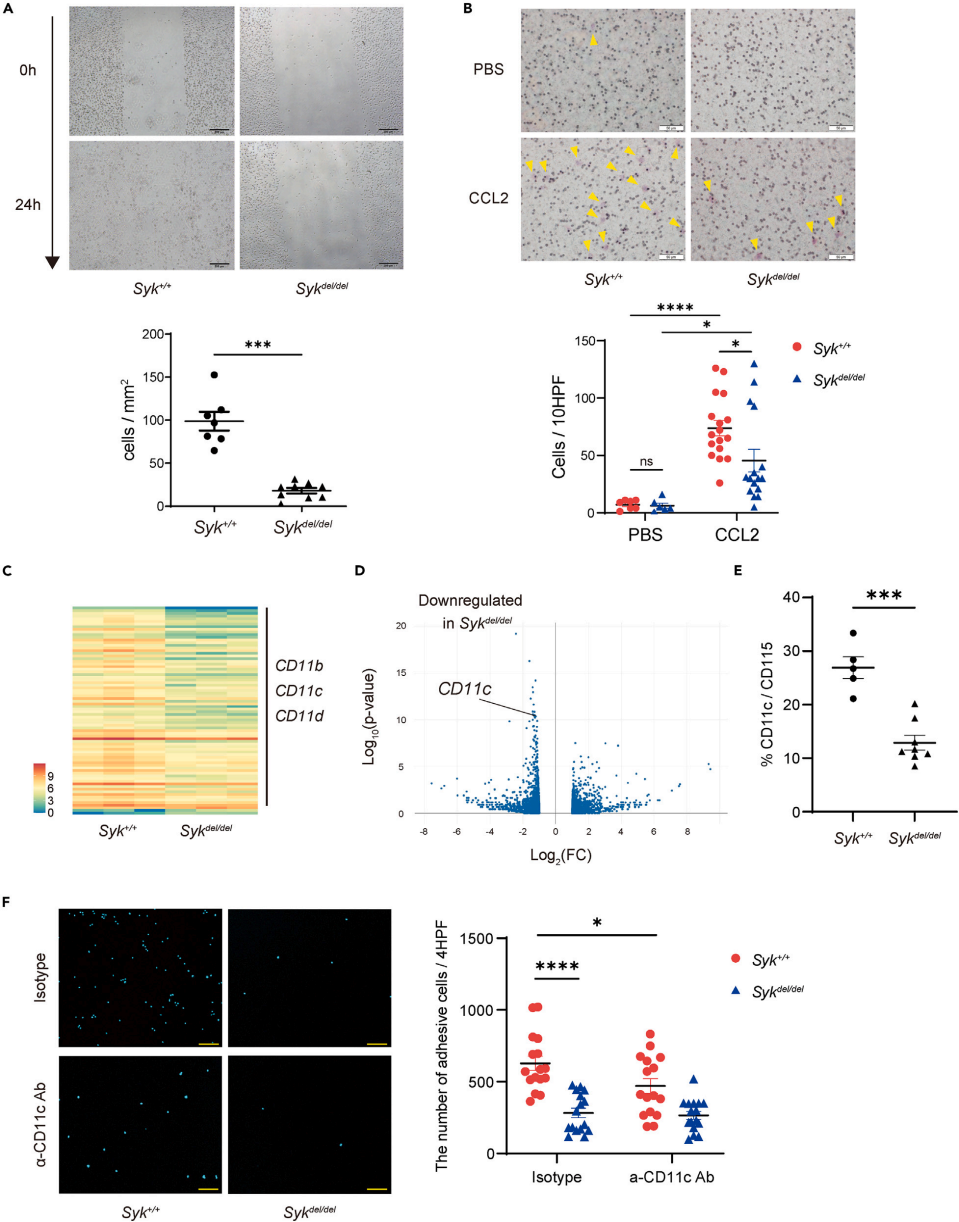
SYK upregulates CD11c expression to enhance cell migration (A) Wound scratch assay: The number of invading cells normalized by the scratch area measured 24 h after the scratch was made. Three independent experiments were performed, and values were averaged based on three fields of view per well (n = 7–9; unpaired *t* test; scale bar: 200 μm). (B) Transwell migration assay: representative microscopic images after hematoxylin-eosin staining. The cell number is a sum of the values of 10 fields of view per well. At least three independent experiments were performed (n = 6–17, one-way ANOVA; Šídák’s multiple comparisons test; arrowheads indicate cells through the membrane. Scale bar: 50 μm). (C and D) RNA-seq was performed with bone marrow monocytes from *Syk*^*+/+*^ and *Syk*^*del/del*^ mice (n = 3 per group). Heatmap representation of the top differentially expressed genes with gradient color bar (C) and Volcano plot (D). (E) CD11c expression on peripheral monocytes isolated from mice fed a high-fat diet for 8 weeks measured using flow cytometry (n = 5–8; unpaired *t* test). Representative gating analyses are shown in Figures S2E and S2F, Cell adhesion assay. (F) Representative microscopic images. The number of adhesion cells stained by DAPI was calculated using confocal microscopy. The cell number is a sum of the values of four fields of view per well. Four independent experiments were performed in quadruplicate wells. Statistical analysis was performed for Isotype-*Syk*^*+/+*^ and CD11c-*Syk*^*+/+*^, and Isotype-*Syk*^*+/+*^ and Isotype-*Syk*^*del/del*^ (n = 16 per group; unpaired *t* test, Mann-Whitney test, respectively; scale bar: 100 μm). Data are shown as mean ± SEM. ∗p < 0.05, ∗∗∗p < 0.001, ∗∗∗∗p < 0.0001; Ns: not significant. CCL2, C-C motif chemokine ligand 2; PBS, phosphate-buffered saline; *Syk*, Spleen tyrosine kinase.

As we determined that *Syk* deletion reduced the macrophage area on the plaque, we hypothesized that *Syk* deletion attenuated cell migration. Therefore, we performed wound scratch and transwell migration assays. We used bone marrow-derived macrophages (BMDMs) and confirmed *Syk* deletion using PCR and western blotting (Figures S2A and S2B). The wound scratch and transwell migration assays showed that *Syk-*deficient BMDMs exhibited decreased cell motility and migration capability owing to C-C motif chemokine ligand 2 (CCL2) (Figures 2A, 2B, and S2C).

To elucidate the cell migration-related genes downstream of *Syk*, we performed a comprehensive gene expression analysis of bone marrow monocytes using RNA sequencing (RNA-seq), which revealed the top 78 differentially expressed genes (DEGs), with a false discovery rate (FDR) < 0.05 (excluding immunoglobulin genes). Downregulated DEGs in *Syk*-deficient monocytes included the migration-related genes *Itgam2* (CD11b isoform), *Itgax* (CD11c), and *Itgad* (CD11d)—also known as integrins (Figures 2C and 2D). Integrins are adhesion molecules that act against endothelial ligands, such as intercellular adhesion molecule 1 (ICAM-1) and vascular cell adhesion molecule 1, in cell migration.^23^^,^^24^^,^^25^ However, *Cd11b*^−/−^ bone marrow-transplanted mice were not protected from atherosclerosis, whereas *Cd11c-* and *Cd11d*-knockout mice exhibited reduced atherosclerosis.^24^^,^^26^^,^^27^ In addition, hypercholesterolemia induced CD11c surface expression on human peripheral monocytes.^28^ Therefore, we focused on *Cd11c* in the context of the downstream signaling of SYK, and *Cd11c* expression was validated using qPCR (Figure S2D). To determine the CD11c protein level, we measured CD11c expression in peripheral monocytes using flow cytometry. CD11c levels decreased significantly on *Syk*-deficient monocytes (p < 0.001), whereas those of CD11b did not decrease (Figures 2E, S2E, and S2F). We then evaluated the adhesive capacity of CD11c against ICAM-1 using an adhesion assay. BMDMs were stimulated with granulocyte-macrophage colony-stimulating factor (GM-CSF) to increase CD11c expression (Figure S2G). The number of adhesive cells in the isotype-control *Syk*-deficient BMDMs decreased more than that in *Syk*-wild type BMDMs. Additionally, the number of adhesive cells in *Syk*-wild type BMDMs incubated with anti-CD11c antibody decreased more than that in *Syk*-wild type BMDMs incubated with the isotype control. CD11c and SYK increased the cell adhesive capacity of BMDMs (Figure 2F).

Collectively, these results suggest that SYK promoted cell migration by regulating *Cd11c* gene and protein expression.

### SYK translocates FOXO1 to the nucleus during CD11c expression

**Figure 3:**
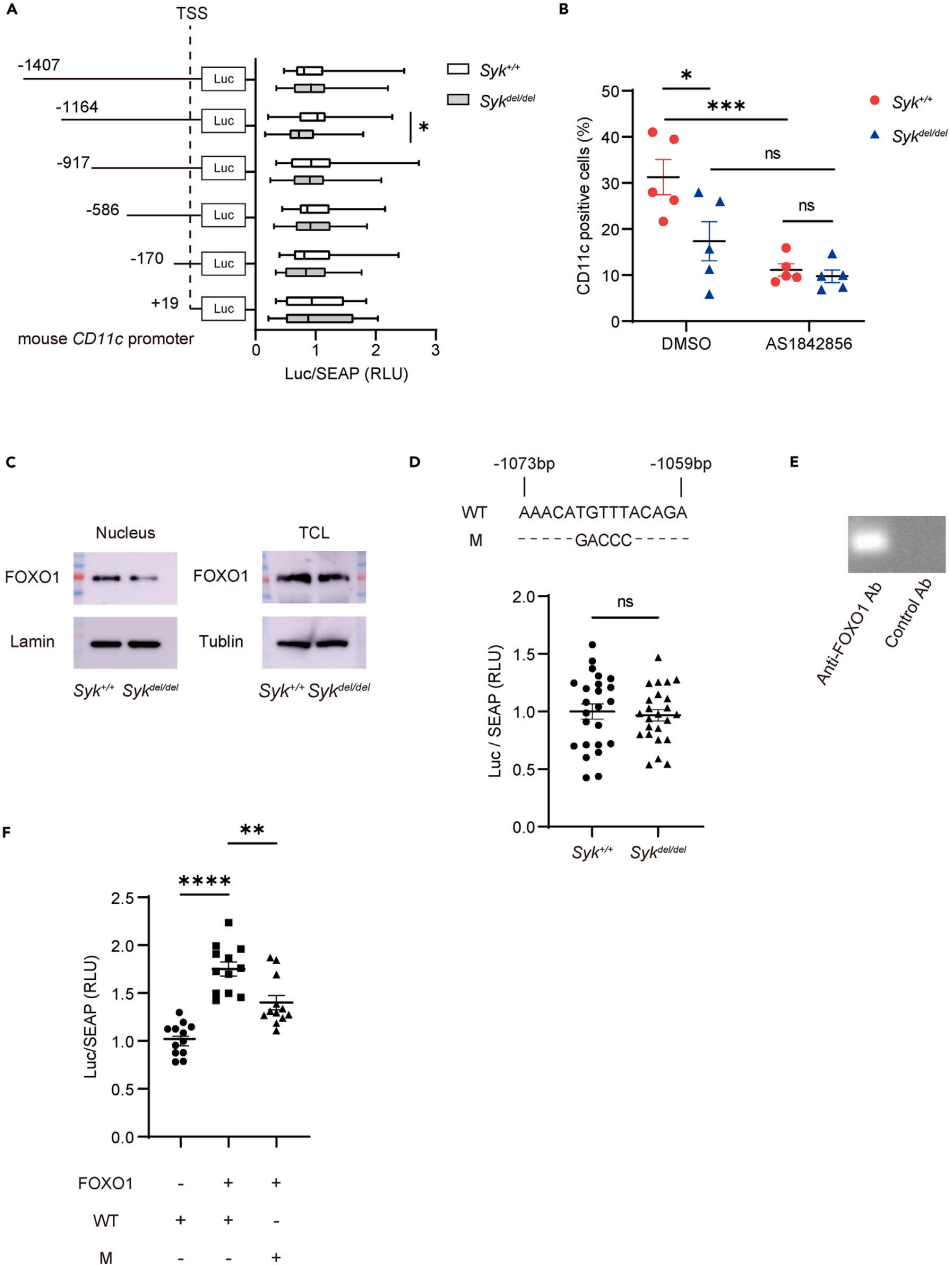
FOXO1 is a transcriptional regulator for CD11c (A) Dual reporter assay revealed the spleen tyrosine kinase (*Syk*)-associated region of the *Cd11c* promoter. Bone marrow-derived macrophages (BMDMs) were infected with a lentivirus vector comprising different lengths of the *Cd11c* promoter, nucleotides encoding Gaussia luciferase (Luc), and secreted embryonic alkaline phosphatase (SEAP). Relative light units (RLU) were normalized with SEAP activity. Six independent experiments were performed in quadruplicate wells (n = 24 per group; Mann-Whitney test). (B) CD11c expression on BMDMs measured using flow cytometry. BMDMs were stimulated overnight with 20 ng/mL of granulocyte-macrophage colony-stimulating factor (GM-CSF) after overnight incubation with a forkhead box protein O1 (FOXO1) inhibitor (58 nM AS1842856). Five independent experiments were performed (n = 5 per group; one-way ANOVA; Šídák’s multiple comparisons test). (C) Western blotting for FOXO1 expression in nuclear fraction and total cell lysate (TCL) of BMDMs. BMDMs were stimulated with 20 ng/mL GM-CSF. Four independent experiments were performed. (D) Dual reporter assay with mutant sequences corresponding to the FOXO1 binding site of the *Cd11c* promoter. Six independent experiments were performed in quadruplicate wells (n = 24 per group; unpaired *t* test). Wild-type (WT) and mutant (M) sequences are indicated above the quantitative graph. (E) ChIP PCR was performed with *Syk*^*+/+*^ cells. Three independent experiments were performed. (F) Dual reporter assay was performed to compare the activity of the WT and mutated (M) *Cd11c* promoter plasmids under FOXO1 overexpression (FOXO1). Three independent experiments were performed in quadruplicate wells (n = 12 per group; one-way ANOVA; Šídák’s multiple comparisons test). See also Figure S3D. Data are shown as mean ± SEM. ∗p < 0.05, ∗∗p < 0.01, ∗∗∗p < 0.001, ∗∗∗∗p < 0.0001; Ns: not significant. TSS = transcription start site.

To determine the region of the *Cd11c* promoter associated with SYK, we performed a luciferase assay using various lengths of the promoter. SYK was associated with a region 917–1,164 bp upstream of the *Cd11c* transcription start site (TSS) (Figure 3A). Next, we determined and sequenced the candidate transcription factors (TFs) that bound 917–1,164 bp upstream of the *Cd11c* TSS using in silico analysis (JASPAR, Table S1).^29^ To identify candidate TFs, we analyzed CD11c expression on BMDMs by adding inhibitors against the TFs using flow cytometry. A forkhead box protein O1 (FOXO1) inhibitor (AS1842856) suppressed CD11c expression (Figure 3B); the inhibitor hampers FOXO1 transcriptional activity.^30^^,^^31^ Additionally, the FOXO1 inhibitor decreased CD11c expression on RAW264.7 cells, a murine macrophage cell line (Figure S3A), and another FOXO1 inhibitor (AS1708727) suppressed BMDM CD11c expression (Figure S3B).

FOXO1 shuttles between the cytosol and nucleus, and intranuclear FOXO1 activates gene expression.^32^ Therefore, the subcellular localization of FOXO1 in GM-CSF-primed BMDMs was evaluated using western blotting. Consistent with the result that the FOXO1 inhibitor suppressed CD11c expression on BMDMs (Figure 3B), the intranuclear FOXO1 levels decreased in *Syk*-deficient BMDMs, whereas the FOXO1 level of total cell lysate remained the same in *Syk*-wild type and *Syk*-deficient BMDMs. These data indicate that SYK affects FOXO1 translocation to the nucleus (Figure 3C). Western blotting revealed the nuclear translocation of FOXO1 in RAW264.7 cells without GM-CSF stimulation (Figure S3C). Then, we evaluated whether FOXO1 binds to the predicted sequence in three ways. First, we mutated the predicted FOXO1 binding sequences present on the reporter plasmid to measure gene expression. The reporter assay performed with the mutated promoter did not show substantial differences between *Syk*-wild type and *Syk*-deficient cells (Figure 3D), whereas a considerable difference was observed between them when performed with the original promoter (Figure 3A). Second, chromatin immunoprecipitation (ChIP) PCR revealed that FOXO1 could bind to the predicted sequence of the *Cd11c* promoter (Figure 3E). Third, FOXO1 overexpression increased *Cd11c* promoter activity, while mutation of the FOXO1 binding site in the *Cd11c* promoter reduced the activity (Figure 3F). The FOXO1 overexpression in 293T cells was confirmed by western blotting (Figure S3D).

These data suggest that SYK induced FOXO1 nuclear translocation, thereby upregulating *Cd11c* transcription.

### JNK controls FOXO1 subcellular localization in SYK-mediated signaling

**Figure 4:**
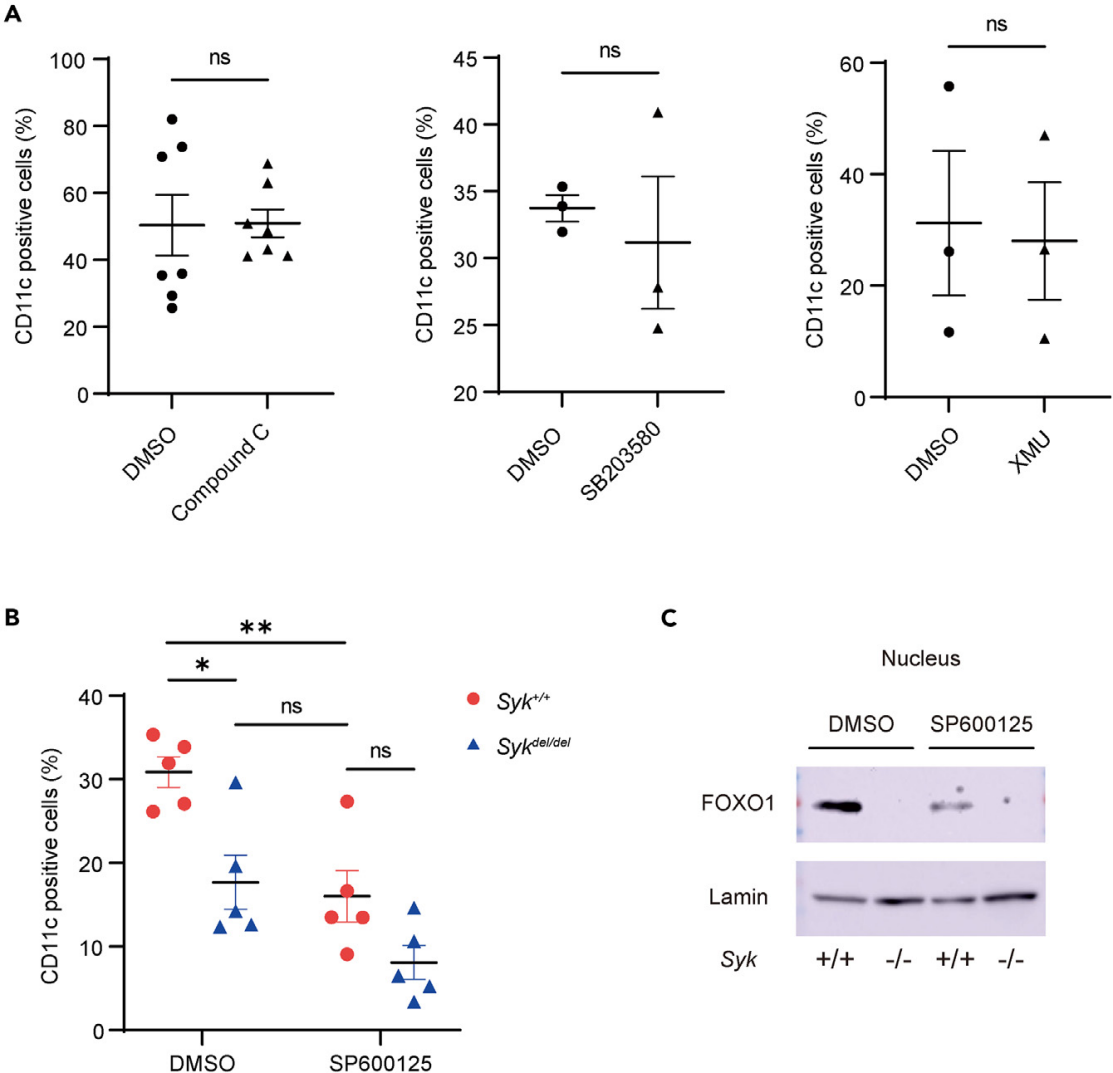
JNK inhibitor suppresses CD11c expression and FOXO1 subcellular localization (A and B) CD11c expression on bone marrow-derived macrophages (BMDMs) measured using flow cytometry. BMDMs were stimulated with 20 ng/mL granulocyte-macrophage colony-stimulating factor (GM-CSF) after overnight incubation with one of the following inhibitors: 500 nM Compound C, 2.65 μM SB203580, 1.92 μM XMU, and 2.25 μM SP600125 (n = 3–7; A, unpaired *t* test and B, one-way ANOVA with Šídák’s multiple comparisons test). (C) Nuclear FOXO1 in GM-CSF-primed BMDMs after the addition of 2.25 μM SP600125 evaluated using western blotting. Three independent experiments were performed. Data are shown as mean ± SEM. ∗p < 0.05, ∗∗p < 0.01; Ns: not significant. Forkhead box O1 = FOXO1; JNK = c-Jun amino-terminal kinase; *Syk* = spleen tyrosine kinase.

We investigated the molecules responsible for mediating FOXO1 translocation-*Cd11c* expression downstream of SYK signaling. Previous studies reported that AMP-activated protein kinase (AMPK), p38, macrophage stimulating 1 (MST1), and c-Jun amino-terminal kinase (JNK) positively control the nuclear translocation of FOXO family proteins.^33^^,^^34^^,^^35^^,^^36^ Thus, we evaluated CD11c expression on GM-CSF-primed BMDMs in the presence of an AMPK inhibitor (Compound C), p38 inhibitor (SB203580), MST1/2 inhibitor (XMU), and JNK inhibitor (SP600125) using flow cytometry. The JNK inhibitor suppressed CD11c expression, whereas the AMPK inhibitor, p38 inhibitor, and MST1/2 inhibitor did not (Figures 4A and 4B). Moreover, the JNK inhibitor suppressed FOXO1 intranuclear translocation (Figure 4C).

Thus, with these data, it is reasonable to conclude that SYK and JNK mediated the GM-CSF receptor/FOXO1/CD11c signaling pathway.

### FOXO1 controls CD11c expression in human cell lines

**Figure 5:**
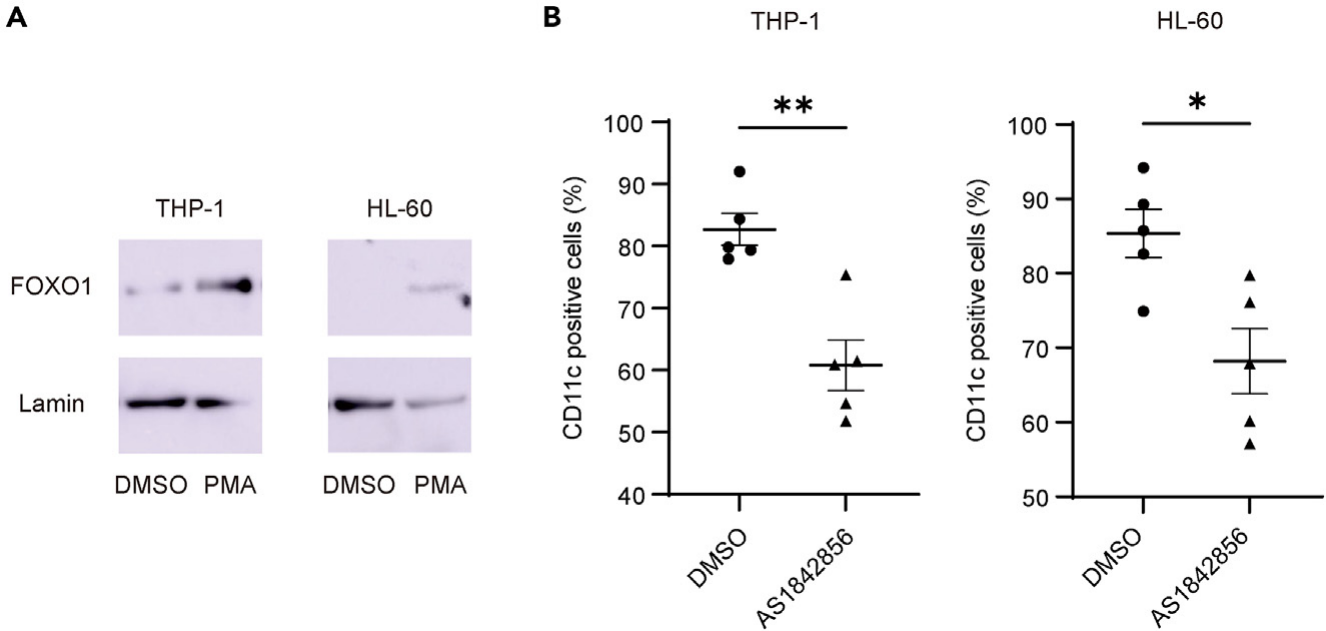
FOXO1 inhibitor suppresses CD11c expression on human monocytes (A) Nuclear FOXO1 levels in phorbol myristate acetate (PMA)-primed THP-1 and HL-60 (1 and 10 ng/mL, respectively; overnight) cells evaluated using western blotting. Three independent experiments were performed. THP-1 cells were incubated with RPMI supplemented with 1% inactivated FBS. (B) CD11c expression on PMA-primed THP-1 and HL-60 cells (1 and 10 ng/mL, respectively; overnight) after overnight incubation with forkhead box protein O1 (FOXO1) inhibitor (58 nM AS1842856) was measured using flow cytometry (n = 5; unpaired *t* test). Data are shown as mean ± SEM. ∗p < 0.05, ∗∗p < 0.01.

The data presented above are based on animal experiments. However, the homology of the FOXO1 binding region in the *Cd11c* promoter between humans and mice is low. To evaluate whether FOXO1 mediated CD11c expression in THP-1 and HL-60 cells (human cell lines), we performed flow cytometry and western blotting. These cell lines, which were differentiated into macrophage/monocyte-like cells with phorbol myristate acetate (PMA), showed upregulated CD11c cell surface expression and PMA-mediated FOXO1 translocation into the nucleus (Figure 5A). Moreover, AS1842856, a FOXO1 inhibitor, suppressed CD11c expression (Figure 5B).

These data indicate that FOXO1 upregulated CD11c expression in both human cell lines and mice.

### FOXO1 inhibitor suppresses CD11c expression and atherosclerosis *in vivo*

**Figure 6:**
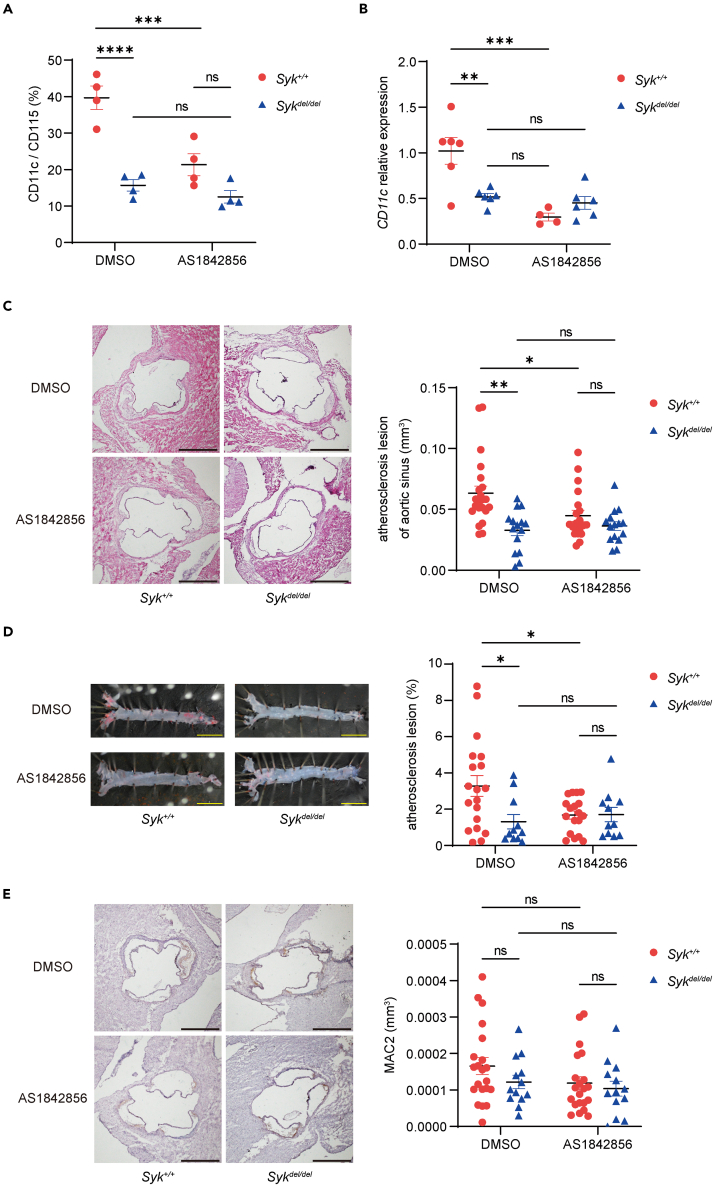
FOXO1 inhibitor suppresses CD11c expression and atherosclerosis *in vivo* (A and B) High-fat-diet-fed mice were intraperitoneally injected with forkhead box protein O1 (FOXO1) inhibitor (20 mg/kg AS1842856) or DMSO (control) for 2 weeks (5 days a week). (A) CD11c cell surface expression in peripheral monocytes measured using flow cytometry (n = 4 per group; one-way ANOVA; Šídák’s multiple comparisons test). (B) *Cd11c* expression in bone marrow monocytes evaluated via qPCR (n = 4–6, one-way ANOVA, Šídák’s multiple). (C–E) Mice fed a high-fat diet were intraperitoneally injected with FOXO1 inhibitor (20 mg/kg of AS1842856) for 8 weeks using the same method as in A and B. (C) Representative hematoxylin-eosin staining images of the aortic sinus (n = 15–22; Kruskal-Wallis test; Dunn’s multiple comparison test; scale bar: 500 μm). (D) Oil Red O staining of the aorta (n = 11–18; one-way ANOVA; Dunnett’s T3 multiple comparisons test; scale bar: 5 mm). (E) Mac-2 staining (macrophage marker) images of the aortic sinus (n = 13–21; Kruskal-Wallis test; Dunn’s multiple comparison test; scale bar: 500 μm). Data are shown as mean ± SEM. ∗p < 0.05, ∗∗p < 0.01, ∗∗∗p < 0.001, ∗∗∗∗p < 0.0001; Ns: not significant. *Syk* = spleen tyrosine kinase.

To confirm FOXO1 function *in vivo*, we determined CD11c expression in peripheral and bone marrow-derived monocytes treated with 20 mg/kg FOXO1 inhibitor AS1842856 for 2 weeks. Flow cytometry and qPCR revealed that AS1842856 suppressed CD11c expression in accordance with the *in vitro* observations (Figures 3B, 6A, and 6B). Finally, we evaluated atherosclerotic development in the presence of 20 mg/kg AS1842856 for 8 weeks. The FOXO1 inhibitor notably suppressed atherosclerosis in the aortic sinus and aorta (Figures 6C and 6D). No substantial difference was observed in the relative abundance of macrophages on atherosclerotic plaques. Nevertheless, the abundance of monocytes isolated from the dimethyl sulfoxide (DMSO) and FOXO1 inhibitor-administered groups of Syk^+/+^ mice decreased (Figure 6E). Moreover, although the levels of LDL-C in Syk^+/+^ mice administered AS1842856 were lower than those in Syk del/del mice administered AS1842856, no considerable differences were observed in the levels of the other serum cholesterols. However, a difference was observed between the DMSO and FOXO1 inhibitor groups of Syk^+/+^ mice in terms of body weight (Figure S4A).

These findings indicate that the FOXO1 inhibitor suppressed mouse atherosclerosis and decreased CD11c expression on monocytes *in vivo*.

## Discussion

Herein, we focused on SYK, a key protein involved in chronic inflammation, and determined that it promoted atherosclerotic development via inflammatory cell migration (Figures 2A, 2B, 2F, and S2C). Additionally, we found that JNK, FOXO1, and CD11c mediated GM-CSF and SYK-related cell migration in monocytes and macrophages. Moreover, AS1842856, a FOXO1 inhibitor, reduced atherosclerosis by decreasing CD11c expression (Figures 6A–6C), indicating its therapeutic potential.

Our finding that *Syk* deletion suppressed atherosclerosis is consistent with a previous study demonstrating that the SYK-specific inhibitor fostamatinib, approved by the FDA for chronic idiopathic thrombocytopenic purpura,^37^ suppressed the development of atherosclerosis in mice.^38^ These results support the application of fostamatinib for the treatment of atherosclerosis. However, further clinical trials in humans are needed. Other studies have also reported that SYK is required for integrin signaling during cell migration.^39^^,^^40^ Here, we observed that SYK upregulated CD11c expression. To evaluate signal transduction between SYK and CD11c *in vitro*, we stimulated BMDMs with GM-CSF to upregulate CD11c expression. GM-CSF plays a role in proinflammatory cytokines in various diseases, and GM-CSF receptor signaling is SYK mediated.^41^^,^^42^ In GM-CSF-knockout mice, atherosclerosis and collagen-induced arthritis are reportedly mitigated.^43^^,^^44^ In humans, otilimab, an anti-GM-CSF monoclonal antibody, ameliorates RA.^45^ The number of GM-CSF-secreting peripheral blood mononuclear cells in SLE increases more than that in healthy controls, positively correlating with anti-double stranded-DNA titers.^46^ With reference to these findings, it is reasonable to suggest that the pathway identified herein contributes to both atherosclerosis and inflammation.

FOXO1 plays various roles, including insulin signaling, lipid metabolism, cellular differentiation, cell proliferation, apoptosis, and DNA repair^47^; however, whether FOXO1 promotes atherosclerosis is unknown. In a previous study, endothelium-specific FOXO isoform (FOXO1, 3a, and 4) triple-knockout mice exhibited atherosclerosis alleviation via enhanced nitric oxide availability, inflammation, and superoxide generation.^48^ In contrast, although myeloid-specific FOXO isoform triple-knockout mice did not exhibit suppression of atherosclerosis, their inflammatory response was increased.^49^ The differences between these reports and our findings suggest that the isoforms function differently because AS1842856 inhibits FOXO1 more specifically than FOXO3A and FOXO4, and analysis showed that the half maximal inhibitory concentration value of AS1842856 for FOXO1 (0.03 μM) is significantly lower than that for FOXO3A and FOXO4 (>1μM).^33^ Although these isoforms have overlapping functions, single-knockout mice express distinct phenotypes;^50^ for instance, atherosclerosis was not suppressed in *FoxO4*-systemic knockout mice.^51^ In addition, *FoxO3* knockdown increases reactive oxygen species production in vascular endothelial cells, contributing to atherosclerotic development.^52^ Although FOXO3 mediates nucleotide-binding oligomerization domain, leucine rich repeat and pyrin domain containing 3 inflammasome activation, which accelerates atherogenesis,^53^ the function of FOXO3 in atherosclerosis remains unclear. Furthermore, the function of each isoform differs in different organs; notably, skeletal muscle-specific FOXO1 overexpression in ApoE^−/−^ mice suppresses atherosclerosis.^54^

The signal transduction pathway from SYK to FOXO1 has been reported: FOXO1 initially binds to a 14-3-3 protein in the cytosol. Stress, including oxidative stress, can activate SYK-mediated JNK signaling, leading to the phosphorylation of 14-3-3 proteins and subsequent dissociation of the FOXO1–14-3-3 protein complex. This results in the translocation of free FOXO1 to the nucleus to upregulate transcription activities.^55^^,^^56^^,^^57^ Taken together, our study and previous reports suggested that the GM-CSF receptor/SYK/JNK/FOXO1/CD11c signaling axis is involved in atherosclerotic development.

Our initial goal was to find a common pathway between atherosclerosis and chronic inflammatory diseases. CD11c, an integrin family member, mediates cell migration via cell-cell adhesion during inflammatory responses.^24^ It is related to the pathogenesis of chronic inflammatory diseases; a previous study reported that *Cd11c* expression in peripheral blood mononuclear cells from patients with RA is higher than that in those from healthy donors and that *Cd11c* levels correlate with serum interleukin-2 (IL)-6 and IL-8 levels.^58^ Furthermore, M1-like macrophages, which express CD11c, contribute to atherosclerosis in SLE.^59^^,^^60^ Anti-integrin monoclonal antibody is approved for the treatment of ulcerative colitis and Crohn disease,^61^ although it does not target CD11c. Additionally, we focused on the common pathway related to Syk and cell migration in atherosclerosis, RA, and SLE. However, there may be additional shared pathways. For instance, the anti-phosphorylcholine antibody, which targets a component of oxidized LDL, exerts an atheroprotective effect.^62^ It has been reported that the lower titers of anti-phosphorylcholine antibodies in individuals with RA and SLE were probably associated with an increased risk of cardiovascular events.^63^^,^^64^ Hence, the capacity to produce anti-phosphorylcholine antibodies might be related to the development of atherosclerosis.

To the best of our knowledge, our study is the first to identify that FOXO1 TF upregulates CD11c expression to induce cell migration in the downstream signaling of SYK. Our findings also suggest that the GM-CSF receptor/SYK/JNK/FOXO1/CD11c signaling axis is commonly involved in the development of atherosclerosis and chronic inflammation. The findings of the study will serve as a reference for future studies that focus on elucidating the role of FOXO1 inhibitors in ameliorating collagen-related diseases in model mice.

### Limitations of the study

Our study has some limitations. First, we hypothesized that *Syk* deletion attenuated cell migration, which could explain decreased macrophage area on atherosclerotic plaques and reduced CD11c expression on monocyte-macrophage lineages in *Syk*^−/−^ mice; however, the decreased macrophage area could also be due to impaired cell differentiation.^22^ The evaluation of cell differentiation and the related genes using RNA-seq (Figures 2C and 2D) could help identify other therapeutic targets. Second, although a downward trend was observed, the administration of FOXO1 inhibitor 5 days a week for 8 weeks did not considerably decrease the macrophage area in atherosclerotic mice that were fed a high-fat diet (Figure 6D). Therefore, a long-term experiment, e.g., 16 weeks, as shown in Figure 1C, could help improve the results. Third, although AS1842856 specifically suppresses FOXO1, it cannot be excluded that the off-target effect of FOXO3a inhibition contributes to atherosclerosis suppression. Additionally, CD11c is mainly expressed in myeloid cells, whereas AS1842856 acts systemically. This difference in the site of action can be elucidated using myeloid-specific *FOXO1*-knockout mice. Fourth, although the signaling axis we identified could be shared by atherosclerosis, RA, and SLE, the plaques in SLE have a tendency of being localized compared to those in general atherosclerosis.^65^ This suggests the presence of distinct atherogenic mechanisms between general atherosclerosis and chronic inflammatory diseases. Further investigation is needed to determine whether the suppression of FOXO1 could alleviate atherosclerosis in SLE patients.

## STAR★Methods

### Key resources table

**Table:** 

REAGENT or RESOURCE	SOURCE	IDENTIFIER
**Antibodies**		

Rabbit polyclonal anti-α-tubulin	Proteintech	Cat#1224-1-AP
Rabbit polyclonal anti-β-actin	Proteintech	Cat#20536-1-AP; RRID:AB_10700003
Rat monoclonal anti-CD115	BioLegend	Cat#135512; RRID:AB_11218983
Rat monoclonal anti-CD11b	BD Bioscience	Cat#557397; RRID:AB_396680
Armenian hamster monoclonal anti-CD11c	Abcam	Cat#ab185783
Armenian hamster monoclonal anti-mouse CD11c	BioLegend	Cat#117308; RRID:AB_313777
Mouse monoclonal anti-human CD11c	BioLegend	Cat#337205; RRID:AB_1236439
Rabbit polyclonal anti-FOXO1	Abcam	Cat#ab39670; RRID:AB_732421
Rabbit monoclonal anti-FOXO1	Cell Signaling Technology	Cat#2880; RRID:AB_2106495
HRP-conjugated Affinipure Goat Anti-Rabbit IgG(H+L)	Proteintech	Cat#SA00001-2; RRID:AB_2722564
Mouse ICAM-1/Fc chimera	BioLegend	Cat#553004
Rabbit polyclonal anti-Lamin B1	Medical and Biological Laboratories	Cat#PM064; RRID:AB_10693917
Rat monoclonal anti-Mac-2	BioLegend	Cat#125401; RRID:AB_1134237
Rabbit anti-rat IgG HRP	Abcam	Cat#ab6734; RRID:AB_955450
Rabbit polyclonal anti-SYK	GeneTex	Cat#GTX100748; RRID:AB_1952121

**Bacterial and virus strains**		

Competent high DH5α	Toyobo Co., Ltd.	Cat#DNA-903

**Chemicals, peptides, and recombinant proteins**		

AS1708727	MedChemExpress	Cat#HY-123046
AS1842856	MedChemExpress	Cat#HY-100596
Blasticidin	Cayman Chemicals	Cat#14499
Compound C	Abcam	Cat#ab120843
Granulocyte–macrophage colony-stimulating factor	Peprotech	Cat#315-03
Phorbol myristate acetate	AdipoGen	Cat#AG-CN2-0010
Polybrene	Nacalai Tesque	Cat #12996-81
Puromycin	Cayman Chemicals	Cat#13884
RBC lysis buffer	pluriSelect	Cat #60-00050-11
SB203580	AdipoGen	Cat#AG-CR1-0030
SP600125	Cayman Chemicals	Cat #10010466
Tamoxifen	Cayman Chemicals	Cat #13258
XMU-MP-1	MedChemExpress	Cat #HY-100526

**Critical commercial assays**		

KAPA2G Fast HotStart ReadyMix	Kapa Biosystems	Cat #KK5610
Peroxidase Stain DAB Kit	Nacalai Tesque	Cat #25985-50
Secrete-Pair™ Dual Luminescence Assay Kit	GeneCopoeia	Cat #LF033
SimpleChIP® Enzymatic Chromatin IP Kit	Cell Signaling Technology	Cat #9002
Thunderbird SYBR qPCR Mix	Toyobo Co., Ltd.	Cat #QPS-201
ReverTra Ace qPCR RT Master Mix	Toyobo Co., Ltd.	Cat #FSQ-201
TaKaRa Bradford Protein Assay Kit	Takara Bio	Cat #T9310A

**Deposited data**		

RNA-Seq	Gene Expression Omnibus	GSE205972

**Experimental models: Cell lines**		

293T	RIKEN Cell Bank	RCB2202
HL-60	RIKEN Cell Bank	RCB3683
PLAT-E	From Prof. Zenichiro Honda, Health Care Center, Ochanomizu University	N/A
RAW264.7	RIKEN Cell Bank	RCB0535
THP-1	RIKEN Cell Bank	RCB1189

**Experimental models: Organisms/strains**		

Mouse: *Syk*^*flox/flox*^	Jackson Laboratory	RRID:IMSR_JAX:017309
Mouse: *Rosa26CreER*^*(T2)+/+*^	Jackson Laboratory	RRID:IMSR_JAX:008463
Mouse: *Ldlr*^*−/−*^	Jackson Laboratory	RRID:IMSR_JAX:002207
Mouse: *Syk*^*flox/flox*^*Rosa26CreER*^*(T2)+/+*^*Ldlr*^*−/−*^	Self-bred	N/A

**Oligonucleotides**		

Primers for PCR analysis of genomic DNA, see Table S2	This paper	N/A
Primers for qPCR analysis, see Table S3	This paper	N/A
Primers for Chi PCR analysis, see Table S4	This paper	N/A

**Recombinant DNA**		

Plasmid: MPRM43508-LvPG04	GeneCopoeia	Cat#MPRM43508-LvPG04

**Software and algorithms**		

ImageJ	National Institute of Health	https://ImageJ.nih.gov/ij/
FlowJo	Becton Dickinson	https://www.flowjo.com/
GraphPad Prism	GraphPad Prism	https://www.graphpad.com/scientificsoftware/prism/
CLC Genomics Workbench	QIAGEN	https://www.qiagen.com/us/products/discovery-and-translational-research/next-generation-sequencing/informatics-and-data/analysis-and-visualization/clc-genomics-workbench

**Other**		

6.5 mm Transwell® with 5.0 μm Pore Polycarbonate Membrane Insert	Corning	Cat #3421
Block Ace	KAC	Cat #UKB80
Blocking One Histo	Nacalai Tesque	Cat #06349-64
Clear Blot Membrane-P Plus	Atto	Cat #WSE-4051
DNeasy Blood & Tissue Kit	QIAGEN	Cat #69504
EasySep Mouse Monocyte Isolation Kit	Veritas	Cat #19861
FuGene®6 Transfection Reagent	Promega	Cat #E2691
KOD-plus-mutagenesis kit	Toyobo Co., Ltd.	Cat #SMK-101
NEBNext Ultra Directional RNA Library Prep Kit	New England Biolabs	Cat #E7420
Nuclear Extraction Kit	Abcam	Cat #ab113474
Clear Blot Membrane-P Plus	Atto	Cat #WSE-4051
ReliaPrep™ RNA Miniprep Systems	Promega	Cat #Z6010
RNA 6000 Pico kit	Agilent Technologies	Cat #5067-1513
rRNA-depletion kit	New England Biolabs	Cat #E6310
Protease Inhibitor Cocktail Set III	Calbiochem	Cat #539134
Tissue-Tek optimal cutting temperature compound	Sakura Finetek	Cat #45833
TRIZOL	Thermo Fisher Scientific	Cat #15596026
Western Diet	Research Diets Inc	Cat #D12079B
Zyppy™ Plasmid Miniprep Kit	ZYMO RESEARCH	Cat #D4036

### Resource availability

#### Lead contact

Further information and requests for resources and reagents should be directed to and will be fulfilled by the lead contact, Hajime Kono (kono@med.teikyo-u.ac.jp).

#### Materials availability

The materials in this article will be shared by the lead contact upon reasonable request.

### Experimental model and study participant details

#### Animals

We obtained *Syk*^*flox/flox*^, *Rosa26CreER*^*(T2)+/+*^, and *Ldlr*^*−/−*^ C57BL/6J mice from the Jackson Laboratory (Bar Harbor, ME, USA). Mice were crossbred to generate *Syk*^*flox/flox*^*Rosa26CreER*^*(T2)+/+*^*Ldlr*^*−/−*^ mice. The *Syk* gene was knocked out using oral administration of 200 mg/kg tamoxifen (13258; Cayman Chemicals, Ann Arbor, MI, USA) for 3 consecutive days at the age of 7–8 weeks. The mice were crossed more than 20 times. Male mice were used for *in vivo* experiments at the indicated age, and 8–15-week-old mice were used for *in vitro* experiments regardless of gender. For atherosclerotic development, mice were fed a high-fat diet (21% fat and 0.21% cholesterol; D12079B; Western Diet; Research Diets Inc., New Brunswick, NJ, USA) from the age of 8 weeks for 2–16 weeks. For FOXO1 inhibition experiments, mice were intraperitoneally injected with 20 mg/kg AS1842856 (HY-100596; MedChemExpress, Monmouth Junction, NJ, USA) 5 days a week for 2–8 weeks. Then, they were euthanized via isoflurane inhalation (Pfizer Inc., New York, NY, USA). The hearts of the euthanized mice were embedded in a Tissue-Tek optimal cutting temperature compound (45833; Sakura Finetek, Tokyo, Japan) after fixation in 10% neutral-buffered formalin; cardiac specimens were cut into 8-μm-thick slices using a microtome (Cryostat; CM 1850; Leica Microsystems GmbH, Wetzlar, Germany). These sections were stained with hematoxylin–eosin and anti-Mac-2 Ab (1:1,000; 125401; BioLegend, San Diego, CA, USA). Subsequently, Mac-2 staining was performed after incubating the sections with 0.3% H_2_O_2_ in methanol to block endogenous peroxidase activity; Blocking One Histo (06349-64; Nacalai Tesque, Kyoto, Japan) was used to minimize non-specific reactions, followed by incubation with rabbit anti-rat immunoglobulin G horseradish peroxidase antibody (1:2,000; ab6734; Abcam, Cambridge, UK). Then, sections were stained using a Peroxidase Stain DAB Kit (25985-50; Nacalai Tesque) and observed under a microscope (BX53; Olympus, Tokyo, Japan). The aortas were longitudinally cut after fixation in 10% neutral-buffered formalin and stained with Oil red O. Representative images were captured using a digital camera (D5200; Nikon, Tokyo, Japan). The stained area of each sample was measured using ImageJ (1.52i; National Institute of Health, Bethesda, MD, USA). Blood was collected from the inferior vena cava using a 24-gauge needle and centrifuged at 800 × *g* for 10 min at 20°C to obtain serum. Serum cholesterol levels were measured using an automated Clinical Chemistry Analyzer (FUJI DRI-CHEM 3500i; Fujifilm, Tokyo, Japan). All animal experiments were approved by the Ethical Committee of Teikyo University School of Medicine, Tokyo, Japan, and the use and care of animals were in accordance with the guidelines of Teikyo University School of Medicine.

#### Cell culture

Bone marrow cells were harvested from the femur and tibia of the mice. BMDMs were generated from the bone marrow cells with RPMI1640, 20% L929 conditioned medium, and 10% fetal bovine serum (FBS), followed by the addition of 5 mL of L929 conditioned medium after 2 days. Adherent cells were regarded as BMDMs. THP-1, 293T, HL-60, and RAW264.7 cells were incubated with RPMI1640 or DMEM and 10% heat-inactivated FBS. In some experiments, cells were starved with RPMI1640 and 1% inactivated FBS. The cells were incubated with the following inhibitors: AS1842856 (HY-100596; Medchem Express), AS1708727 (HY-123046; MedChemExpress), Compound C (ab120843; Abcam), SB203580 (AG-CR1-0030; AdipoGen, San Diego, CA, USA), SP600125 (10010466; Cayman Chemicals), and XMU-MP-1 (HY-100526; MedChemExpress). PLAT-E cells were incubated with DMEM, 10% heat-inactivated FBS, 1 μg/mL puromycin (13884; Cayman Chemicals), and 10 μg/mL blasticidin (14499; Cayman Chemicals).

### Method details

#### PCR analysis using genomic DNA

The genomic DNA of murine organs and primary cells was extracted using a DNeasy Blood & Tissue Kit (69504; Qiagen, Hilden, Germany). Next, 20 μL of PCR reaction mixture containing 10 ng of the template DNA, KAPA2G Fast HotStart ReadyMix with dye (2×) (KK5610; Kapa Biosystems, Wilmington, MA, USA), 0.5 μM forward primer, and 0.5 μM reverse primer was prepared. The PCR programs were as follows: for *Syk*, the first step was at 94°C for 3 min, followed by 35 cycles with denaturation at 94°C for 30 s, annealing at 65°C for 30 s, and extension at 72°C for 60 s; for *Ldlr* WT, the first step was at 95°C for 3 min, followed by 33 cycles with denaturation at 94°C for 30 s, annealing at 52°C for 45 s, and extension at 72°C for 50 s; and for *Ldlr* knockout, the first step was at 95°C for 3 min, followed by 35 cycles with denaturation at 94°C for 30 s, annealing at 53°C for 45 s, and extension at 72°C for 70 s. The primer sequences are provided in Table S2.

#### Western blotting

Proteins were extracted from tissues or cultured cells using lysis buffer (20 mM Tris-HCL, 150 mM NaCl, 0.5% Triton X-100, 1 mM ethylenediamine tetraacetic acid [EDTA], Protease Inhibitor Cocktail Set III 1:200 [539134; Calbiochem, Gibbstown, NJ, USA], and 0.5% sodium deoxycholate, pH 7.4) or a Nuclear Extraction Kit (ab113474; Abcam). The tissue and nuclear fractions were homogenized and sonicated, respectively. The obtained protein was quantified using a TaKaRa BCA Protein Assay Kit (T9300A; Takara Bio, Shiga, Japan) or TaKaRa Bradford Protein Assay Kit (T9310A; Takara Bio) according to the manufacturer’s instructions. Protein lysates were then incubated with SDS sample buffer, boiled at 95°C for 5 min, separated using 10% SDS-PAGE, and transferred to PVDF membranes (WSE-4051; Clear Blot Membrane-P Plus; Atto, Tokyo, Japan). Membranes were blocked overnight with 4% Block Ace (UKB80; KAC Co. Ltd., Kyoto, Japan) at 4°C and incubated with primary antibodies at 4°C overnight, followed by overnight incubation with HRP-conjugated AffiniPure Goat Anti-Rabbit IgG (H+L) (1:10,000 dilution; SA00001-2; Proteintech, Rosemont, IL, USA) at 4°C. Images were obtained using an AI 680 Imager (GE Healthcare Life Sciences, Chicago, IL, USA). Antibodies were then diluted in Immunoreaction Enhancer Solution (NKB-401; Can Get Signal; Toyobo Co., Ltd., Osaka, Japan). Primary antibodies used in this experiment were as follows: anti-Syk Ab (1:2,000; GTX100748; GeneTex, Alton Pkwy Irvine, CA, USA), anti-FOXO1 Ab (1:1,000; C29H4, 2880; Cell Signaling Technology, Danvers, MA, USA), anti-β-actin Ab (1:5,000; 20536-1-AP; Proteintech), anti-α-tubulin Ab (1:5,000; 11224-1-AP; Proteintech), and anti-Lamin B1 Ab (1:2,000; PM064; Medical and Biological Laboratories, Tokyo, Japan).

#### Wound scratch assay

BMDMs were incubated in 24-well plates (4 × 10^5^ cells/well) overnight. Cell-free zones were made using 200-μL tips and, after 24 h, the number of cells invading the cell-free zone was calculated using ImageJ 1.52i. Additionally, the BMDMs were cultured in 8-well glass chamber slides (2 × 10^5^ cells/well) overnight. After the cell-free zone was made (as described above), cells were observed under a confocal microscope (FV10i; Olympus) every 15 min for 24 h. The distance traveled by the cells was measured using ImageJ 1.52i.

#### Transwell migration assay

BMDMs were detached with 0.1% *w*/v trypsin and EDTA after pre-incubation with RPMI1640 and 0.5% FBS for 2 h. The BMDMs (4 × 10^4^ cells/well) were seeded in the upper side of the transwell in a 24-well plate (3421; pore size 5 μm; Corning, Kennebunk, ME, USA) for 10 min. Then, RPMI1640 supplemented with 0.5% FBS and 100 ng/mL CCL2 was added to the lower chamber, and BMDMs were incubated at 37°C for 3 h. The upper side of the membrane was wiped, fixed with 70% ethanol, and stained with hematoxylin–eosin. Adherent cells on the underside were counted using a microscope (BX53; Olympus). The cell number is expressed as the sum of cell counts in 10 fields of view per well.

#### qPCR and transcriptomic analysis

Bone marrow monocytes were isolated using an EasySep Mouse Monocyte Isolation Kit (19861; Veritas, Santa Clara, CA, USA). The total RNA was purified using ReliaPrep RNA Miniprep Systems (Z6010; Promega, Madison, WI, USA), followed by a reverse transcription reaction to obtain cDNA using ReverTra Ace qPCR RT Master Mix (FSQ-201; Toyobo Co., Ltd.) according to the manufacturer’s protocols. qPCR was performed using Thunderbird SYBR qPCR Mix (QPS-201; Toyobo Co., Ltd.) according to the manufacturer’s protocol in a 7500 FAST Real-Time PCR system (Applied Biosystems, Foster City, MA, USA). The number of amplification cycles was 40. The relative expression levels were calculated using the ΔΔCt method. The primers are listed in Table S3. For transcriptomics analysis, total RNA was isolated using TRIZOL (15596026; Thermo Fisher Scientific, Waltham, MA, USA). RNA quality was analyzed using an RNA 6000 Pico Kit (5067-1513; Agilent Technologies, Santa Clara, CA, USA). An RNA-seq library was constructed from 500 ng of total RNA using an NEBNext Ultra Directional RNA Library Prep Kit (E7420; New England Biolabs, Ipswich, MA, USA) after treatment with the rRNA-depletion kit (E6310; New England Biolabs). Then, paired-end sequencing was performed using NextSeq500 (Illumina, San Diego, CA, USA) by Tsukuba i-Laboratory LLP (Tsukuba, Ibaraki, Japan). Sequence reads were mapped into the mouse reference genome assembly (GRCm38/mm10) and quantified for annotated genes using the CLC Genomics Workbench (QIAGEN). The expression levels were normalized in transcripts per million. DEGs were defined as genes with FDR-corrected p < 0.05. The datasets are available at Gene Expression Omnibus (GSE205972).

#### Flow cytometry analysis

BMDMs were detached using 0.1% *w*/*v* trypsin and EDTA, and trypsin digestion was stopped using phosphate-buffered saline (PBS) (-) and 10% FBS. PMA (AG-CN2-0010; AdipoGen)-primed THP-1 and HL-60 cells were detached using cold PBS (-). Peripheral blood cells drawn from the inferior vena cava were incubated thrice with RBC lysis buffer (60-00050-11; pluriSelect, Leipzig, Germany) for 2 min to remove red blood cells. These samples were stained with Abs diluted in PBS (-) for 20 min on ice, followed by washing with 1 mL of PBS (-), centrifugation at 400 × *g* for 5 min at 4°C, and resuspension in 200 μL of PBS (-) before analysis. Data were acquired on a flow cytometer (EC800; Sony, Tokyo, Japan). Graphs were created using FlowJo (10.8.1; Becton Dickinson, Franklin Lakes, NJ, USA). The antibodies used in this experiment were anti-CD115 Ab (1:500; 135512; BioLegend), anti-murine CD11c (1:400; 117308; BioLegend), anti-human CD11c (1:100; 337205; BioLegend), and anti-CD11b (1:400; 557397; BD Biosciences, San Diego, CA, USA).

#### Cell adhesion assay

For this experiment, 96-well flat-culture plates were coated overnight with 2 μg/mL ICAM-1/Fc chimera (553004; BioLegend) at 4°C. The vessel was blocked with 2% bovine serum albumin for 1 h at 37°C and washed with PBS (-). Before the experiments, BMDMs were primed with 5 ng/mL GM-CSF (315-03; PeproTech, Inc., Rocky Hill, NJ, USA) for 2 days for CD11c upregulation. BMDMs were harvested with 0.1% w/v trypsin and EDTA, which was blocked with 50 μg/mL anti-CD11c (ab185783; Abcam) for 30 min on ice. Then, they were washed and resuspended in warm DMEM to obtain a concentration of 1 × 10^5^ cells/well. BMDMs were incubated in the ICAM-1/Fc chimera-coated plate at 37°C for 10 min. Finally, BMDMs were washed and fixed with 70% ethanol, followed by DAPI staining. The numbers of adherent cells were counted automatically via confocal microscopy (CQ1; Yokogawa, Tokyo, Japan).

#### CD11c reporter assay

Lentivirus plasmids carrying the *Cd11c* promoter sequence and nucleotide sequences encoding Gaussia luciferase and secreted embryonic alkaline phosphatase (SEAP) for normalization were obtained from GeneCopoeia (MPRM43508-LvPG04; Rockville, MD, USA). We generated various lengths of the *Cd11c* promoter and mutation sequence (5′-GACCC-3′; 1069–1065 bp upstream of the *Cd11c* TSS) using the KOD-Plus-Mutagenesis Kit (SMK-101; Toyobo Co., Ltd.); plasmids were transfected into competent high DH5α (DNA-903; Toyobo Co., Ltd.) and purified using a Zyppy Plasmid Miniprep Kit (D4036; Zymo Research, Irvine, CA, USA). Sequences were analyzed using a DNA sequencer at Eurofins Genomics (Luxembourg City, Luxembourg). The lentiviral plasmids were then transfected to PLAT-E using FuGene6 Transfection Reagent (E2691; Promega) and the supernatants, including the lentivirus, were collected. The supernatant with 8 μg/mL polybrene (12996-81; Nacalai Tesque) was added to BMDMs and centrifuged at 1000 × *g* at 32°C for 1 h. The supernatants were collected 2 days after from the day of infection. For FOXO1 overexpression, 293T cells were transfected with 50 ng of plasmids containing the CMV promoter and murine FOXO1 coding sequence and 50 ng of plasmids containing either the original or mutated *Cd11c* promoter, Gaussia luciferase, and SEAP using FuGene6 Transfection Reagent (E2691; Promega). The supernatants were collected 2 days after the day of transfection. Luciferase and SEAP activities were measured using a Secrete-Pair Dual Luminescence Assay Kit (LF033; GeneCopoeia) and luminometer (SpectraMax i3; Molecular Devices, San Jose, CA, USA), as per the manufacturer’s instructions.

#### ChiP PCR

DNA was purified using a SimpleChIP Enzymatic Chromatin IP Kit (9002; Cell Signaling Technology) and anti-FOXO1 antibody (ab39670; Abcam), as per the manufacturer’s protocols, after which PCR was performed. BMDMs were fixed with 1% formaldehyde for 30 min at 20°C, and the reaction was stopped using glycine. Subsequently, BMDMs were collected and incubated in wash buffers A and B with micrococcal nuclease at 37°C for 20 min; the reaction was stopped using EDTA. The suspension was sonicated five times for 10 s. After centrifugation at 9400 × *g* for 10 min at 4°C and the addition of ChiP buffer, the suspension was incubated overnight with 1 μg of anti-FOXO1 antibody (ab39670; Abcam) and Normal Rabbit IgG (2729; Cell signaling Technology) at 4°C, followed by incubation with 30 μL of ChIP-Grade Protein G Agarose Beads (9007; Cell Signaling Technology) at 4°C for 2 h. After centrifugation, the supernatant was discarded, and the pellet was incubated with ChiP elution buffer at 65°C for 30 min. After the supernatants were collected, DNA was purified. PCR was performed with 20 μL of PCR reaction mixture containing 2 μL of the purified DNA, KAPA2G Fast HotStart ReadyMix with dye (2×) (KK5610, Kapa Biosystems), 0.5 μM forward primer, and 0.5 μM reverse primer. The primer sequences are provided in Table S4.

### Quantification and statistical analysis

All data were analyzed using GraphPad Prism (version 9; GraphPad Software, San Diego, CA, USA); values are expressed as mean ± SEM. Normal distribution analysis was performed using the Shapiro–Wilk test or D’Agostino and Pearson test. For two groups, an unpaired *t*-test (parametric data) or Mann–Whitney *U* test (nonparametric data) was used. For more than two groups, one-way analysis of variance (ANOVA) with Dunnett’s T3 multiple comparisons test, Šídák’s multiple comparisons test (parametric data), or Kruskal–Wallis test in conjunction with Dunn’s multiple comparisons test (nonparametric data) was used. Significant differences were defined by two-tailed p < 0.05 or multiplicity-adjusted p < 0.05. The asterisks are defined as follows: ∗, p < 0.05; ∗∗, p < 0.01; ∗∗∗, p < 0.001; and ∗∗∗∗, p < 0.0001.

## Data Availability

• The RNA-seq data have been deposited at Gene Expression Omnibus (GSE205972) and are publicly available as of the date of publication. • This paper does not report original code. • Any additional information required to reanalyze the data reported in this paper is available from the lead contact upon request.
