# Supplementary material for: GM-CSF receptor/SYK/JNK/FOXO1/CD11c signaling promotes atherosclerosis
Source: iScience. 2023 Jul 11;26(8):107293. doi: 10.1016/j.isci.2023.107293 (PMC10382675; doi:10.1016/j.isci.2023.107293)
Supplement: Document S1. Figures S1–S4 and Tables S1–S4 [file mmc1.pdf]

iScience, Volume *26*

Supplemental information

GM-CSF receptor/SYK/JNK/FOXO1/CD11c signaling promotes atherosclerosis

Daisuke Tsukui, Yoshitaka Kimura, and Hajime Kono

iScience, Volume ■ ■

Supplemental information

GM-CSF receptor/SYK/JNK/FOXO1/CD11c signaling promotes atherosclerosis

Daisuke Tsukui, Yoshitaka Kimura, and Hajime Kono

Supplemental information

Table S1. List of predicted transcription factors, related to Figure 3

Matrix ID	Name	Score	Relative score	Start	End	Strand	Predicted sequence
MA0480.1	Foxo1	11.675	0.919932	-1071	-1061	+	acatgtttaca
MA0739.1	Hic1	11.525	0.953161	-958	-950	+	atgccagct
MA0047.2	Foxa2	11.3912	0.904454	-1068	-1057	+	tgtttacagaaa
MA0829.1	Srebf1(var.2)	10.9087	0.902402	-1126	-1117	+	atcacctgag
MA0816.1	Ascl2	10.242	0.884744	-926	-917	+	ggctgctgct
MA0851.1	Foxj3	10.2088	0.830502	-1004	-988	+	agcaggtcaacatagg a
MA0151.1	Arid3a	9.84424	1	-1077	-1072	+	attaaa
MA0614.1	Foxj2	9.77521	0.923773	-999	-992	+	gtcaacat
MA0467.1	Crx	9.74681	0.882589	-991	-981	+	aggaagatttg
MA0079.2	SP1	9.64527	0.874205	-1102	-1093	+	cccagcccca
MA0854.1	Alx1	9.1537	0.808741	-955	-939	+	ccagctacttagcaccc
MA0739.1	Hic1	8.95423	0.899905	-1026	-1018	+	atgccatca
MA0158.1	HOXA5	8.82647	0.963737	-963	-956	+	cacaaatg
MA0124.2	Nkx3-1	8.63568	0.883942	-952	-944	+	gctacttag
MA0158.1	HOXA5	8.41119	0.949112	-1062	-1055	+	cagaaatg
MA0145.2	Tcfcp2l1	8.38124	0.853169	-939	-926	+	ccagttctttgctg
MA0160.1	NR4A2	8.24213	0.880658	-1002	-995	+	caggtcaa
MA0498.1	Meis1	8.18814	0.837151	-969	-955	+	tactgtcacaaatgc
MA0840.1	Creb5	7.95934	0.857912	-1132	-1121	+	tatgatatcacc
MA1620.1	Ptf1a(var.3)	7.9295	0.843334	-1127	-1116	+	tatcacctgagt

This file was downloaded from JASPAR (https://jaspar.genereg.net/faq/) on Sep 28, 2021. Predicted transcription factors (TFs) that could bind to the SYK-related *Cd11c* promoter region were analyzed. The results show top 20 TFs binding to positive strands and are sorted by score.

Table S2. Primers for PCR analysis of genomic DNA, related to STAR Methods

Primer	Sequence (5′ - 3′)
Syk forward	GCCCGTTCTGTGCCTACTGG
Syk reverse	GCTGGTTCCCTTTTCCTTCC
Ldlr common forward	AGGATGACTTCCGATGCCAG
Ldlr knockout reverse	AGGTGAGATGACAGGAGATC
Ldlr WT reverse	GCAGTGCTCCTCATCTGACTTG

Table S3. Primers used for qPCR, related to STAR Methods

Primer	Sequence (5′ - 3′)
Cd11c forward	CTGGATAGCCTTTCTTCTGCTG
Cd11c reverse	GCACACTGTGTCCGAACTCA
GAPDH forward	AGGTCGGTGTGAACGGATTTG
GAPDH reverse	TGTAGACCATGTAGTTGAGGTCA

Table S4. Primers for ChiP PCR, related to STAR Methods

Primer	Sequence (5′ - 3′)
Chip PCR forward	TAAAGTCCCAGCCCCAAG
Chip PCR reverse	TGGCATTTGTGACAGTAC

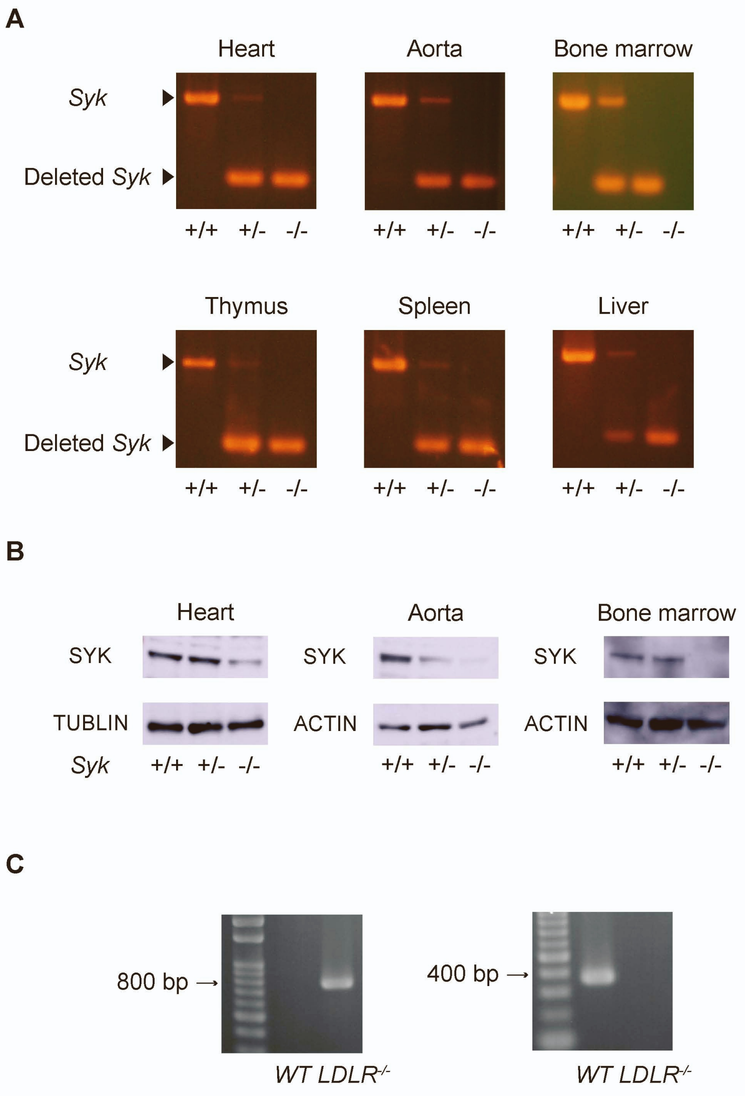

Figure S1. Analysis of *Syk* and *Ldlr* knockout, related to Figure 1

PCR analyses and western blotting show deletion of *Syk* gene (A) and protein (B) from several organs, respectively. (C) *Ldlr* knockout was analyzed using PCR. Images were obtained from at least three independent experiments.

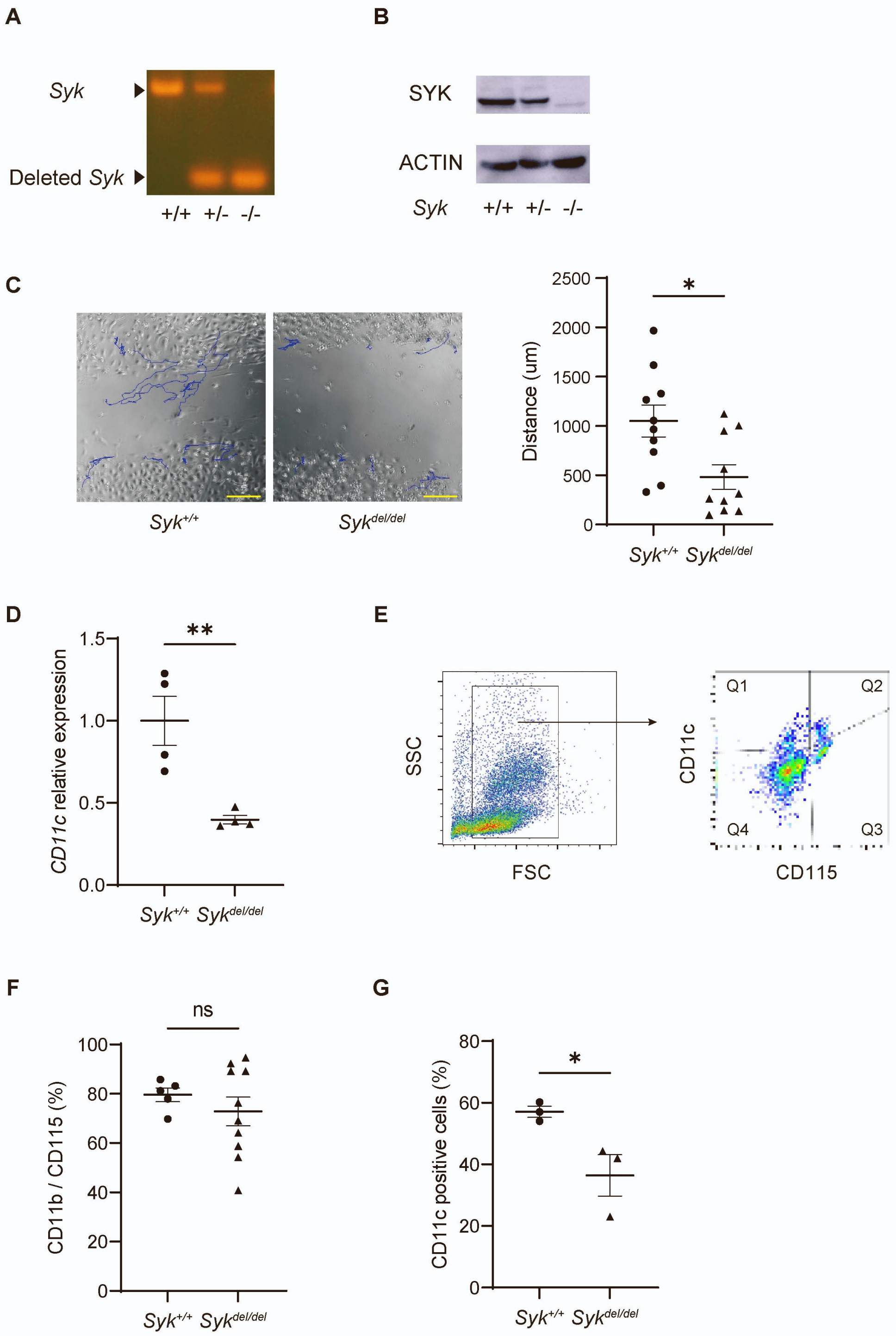

Figure S2. CD11c and CD11b expression downstream of SYK, related to Figure 2

PCR analysis and western blotting show deletion of spleen tyrosine kinase (*Syk*) gene (A) and protein (B) in bone marrow-derived macrophages (BMDMs). Images were obtained from three independent experiments. (C) Pictures of individual cell migration events were serially captured every 15 min for 24 h and combined into one. The moving distance of 10 individual cells was tracked and measured by ImageJ1.52i (unpaired t-test). (D) *Cd11c* expression was measured using qPCR. Total RNA was extracted from bone marrow monocytes from mice fed a high-fat diet for 2 weeks (n = 4 per group; unpaired t-test). (E) Flow cytometry gating analysis for peripheral monocytes. (F) CD11b expression on peripheral monocytes isolated from mice fed a high-fat diet for 8 weeks measured using flow cytometry. Representative gating analyses (n = 5–10; unpaired t-test). (G) CD11c expression on BMDMs measured using flow cytometry (n = 3 per group; unpaired t-test). BMDMs were stimulated overnight with 5 ng/mL granulocyte-macrophage colony-stimulating factor (GM-CSF). Data are shown as mean ± SEM. * $P < 0.05$, ** $P < 0.01$; Ns: not significant. FSC = forward scatter; SSC = side scatter.

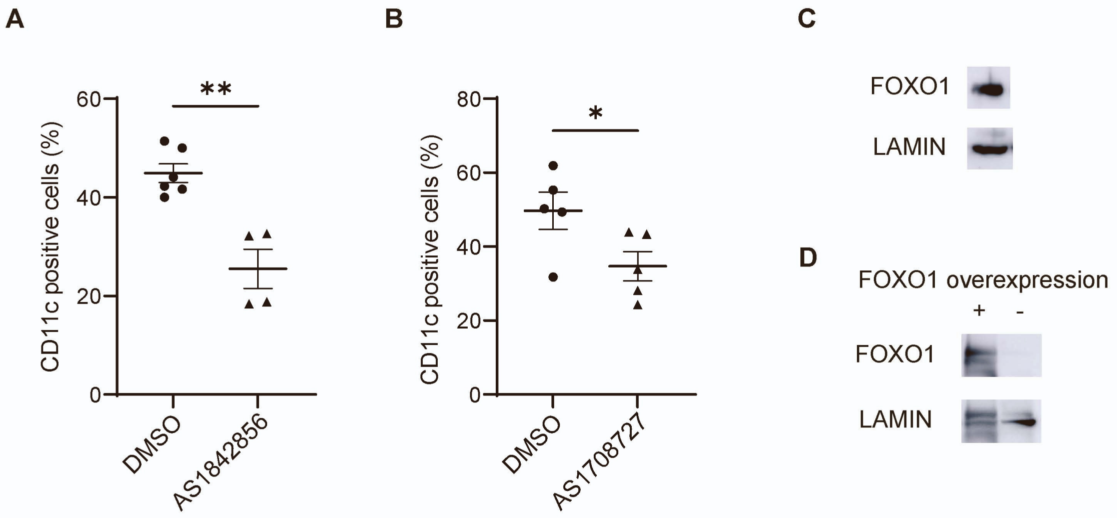

Figure S3. AS1842856 inhibits CD11c expression in murine cell lines, related to Figure 3
(A) CD11c expression on RAW264.7 cells measured using flow cytometry after overnight incubation with Forkhead box O1 (FOXO1) inhibitor (58 nM AS1842856) (n = 4–6 per group; Mann–Whitney test). (B) CD11c expression of bone marrow-derived macrophages (BMDMs) of *Syk*$^{+/+}$ measured using flow cytometry. The BMDMs were stimulated overnight with 20 ng/mL granulocyte-macrophage colony-stimulating factor (GM-CSF) after overnight incubation with another FOXO1 inhibitor (2.82 μM AS1708727). Five independent experiments were performed (n = 5 per group; unpaired t-test). (C) Representative data of western blotting analysis of FOXO1 in the nuclear fraction of RAW264.7 cells. Three independent experiments were performed. (D) Western blotting analysis of FOXO1 in the nuclear fraction of 293T cells transfected with FOXO1 overexpression plasmid. At least three independent experiments were performed. Data are shown as mean ± SEM. * $P < 0.05$, ** $P < 0.01$.

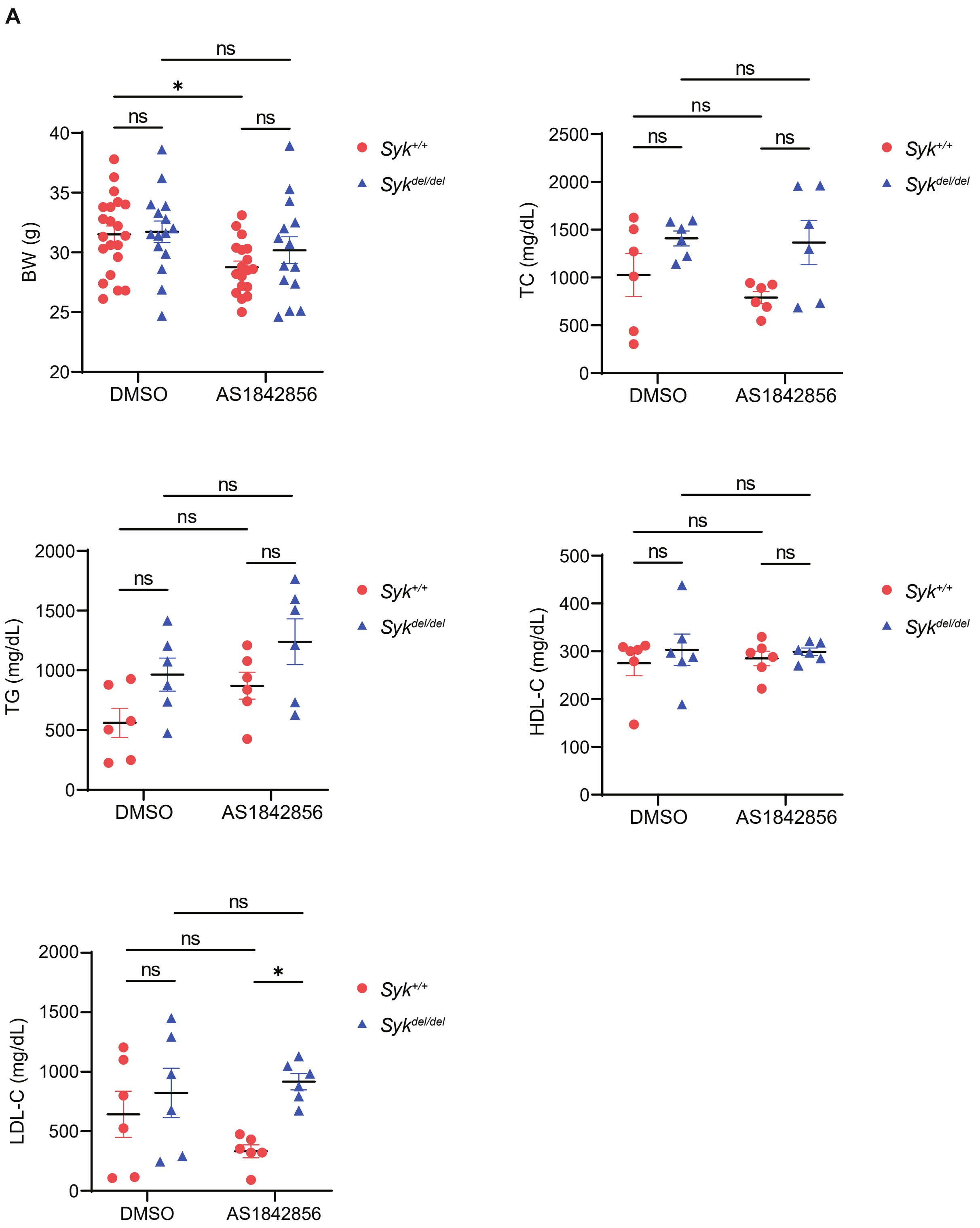

Figure S4. Characteristics of atherosclerosis-prone mice treated with FOXO1 inhibitor, related to Figure 6 (A) Each graph shows body weight (BW), serum levels of total cholesterol (TC), triglyceride (TG), HDL-cholesterol (HDL-C), and LDL-cholesterol (LDL-C) (n = 14–21 for BW; n = 6 per group for other parameters; one-way ANOVA followed by Dunnett’ s T3 multiple comparisons test for BW and LDL-C, one-way ANOVA followed by Šídák’ s multiple comparisons test for TC and TGs, and Kruskal–Wallis test followed by Dunn’ s multiple comparisons test for HDL-C were performed. Data are shown as mean ± SEM. * $P < 0.05$; Ns: not significant. *Syk* = spleen tyrosine kinase. Forkhead box O1 = FOXO1.
